# SOCIAL NETWORKS, HEALTH, AND WELL-BEING AMONG PEOPLE LIVING WITH DEMENTIA

**DOI:** 10.1093/geroni/igz038.277

**Published:** 2019-11-08

**Authors:** Eleanor S McConnell, Kirsten Corazzini, T Robert Konrad

**Affiliations:** 1 Duke University, Durham, North Carolina, United States; 2 University of North Carolina at Chapel Hill, Chapel Hill, North Carolina, United States

## Abstract

Although the impact of dementia on the health and well-being of those living with Alzheimer’s Disease and related Disorders (ADRD) and their care partners has been widely studied, less attention has been paid to how the disease impacts individuals within the context of their larger social networks. This symposium presents findings from a series of integrated studies aimed at strengthening measurement of health and well-being among older adults with living with dementia and well-being among members of their social networks. Findings will be presented from five studies: (1) a scoping review of social network measurement in older adults in chronic illness, including dementia, that emphasizes the use of technology in measuring older adults’ social networks; (2) a simulation study to evaluate the feasibility and reliability of sensor technology to measure social interaction among a person living with dementia and others in their immediate surroundings; (3) development of a web-based application that allows older adults to map and activate their social networks; (4) a qualitative analysis of interviews from persons living with dementia, their unpaid caregivers, and paid caregivers from an adult day health program concerning well-being focused outcomes; and (5) a mixed methods analysis of the feasibility of using both traditional and novel measures of health and well-being deployed among networks of people living with dementia. Emerging technologies for measuring social networks health and well-being hold promise for advancing the study of the relationship-based nature of care for people living with dementia.

